# End-of-life considerations in the ICU in Japan: ethical and legal perspectives

**DOI:** 10.1186/2052-0492-2-9

**Published:** 2014-02-18

**Authors:** Jun Makino, Shigeki Fujitani, Bridget Twohig, Steven Krasnica, John Oropello

**Affiliations:** Department of Critical Care Medicine, Mount Sinai Medical Center, One Gustave L. Levy Place, Box 1264, New York, NY 10029 USA; Clinical Professor of Emergency/Critical Care Medicine Department, St. Marianna Medical School, 2-16-1 Sugao Miyamae-ku, 216-8511 Kawasaki City, Kanagawa, Prefecture, Japan

**Keywords:** End-of-life, ICU, Japan, USA, East, Asian countries, Palliative care, Advance directive, Living will

## Abstract

In Japan, the continuation of critical care at the end of life is a common practice due to the threat of legal action against physicians that may choose a palliative care approach. This is beginning to change due to public debate related to a series of controversial incidents concerning end-of-life care over the last decade. In this review we contrast and compare the history and evolution of end-of-life care in Japan vs. the USA and other Asian countries. Efforts by the Japanese Society of Intensive Care Medicine (JSICM) to establish better end-of-life care systems, as well as future directions in palliative care in Japan, are discussed.

## Introduction

Through advances in medicine, patients with severe acute and chronic diseases are now surviving longer, and end-of-life care issues occur with greater frequency in the Intensive Care Unit (ICU).

The Japanese National Institute of Population and Social Security Research recently estimated that the population aged ≧65 years will increase from 29.48 million to 34.64 million by 2060 [1]. In addition, the Organization for Economic Cooperation and Development (OECD) reported that the 2.2:1,000 doctor-patient ratio in Japan is lower than in other industrialized countries (Table 1) [2].

**Table 1:** **International comparison of the ratio of physicians to patients and the number of ICU beds**

Country	Ratio of physicians to patients (per 1,000 population)[2]	Number of ICU beds (per 100,000 population)[4, 5]
Germany	3.7	24.6
France	3.3	9.3
England	2.7	3.3
USA	2.4	20
Canada	2.4	13.5
Japan	2.2	4 to 5

Looking specifically at the critical care in Japan, only 822 (11%) of the 7,530 facilities nationwide have ICUs with 6,530 ICU beds nationwide [3]. Furthermore, the estimated ratio of ICU beds per 100,000 people is only 4 to 5 in Japan [4], a ratio that lags in comparison to Europe and North America (Table 1) [5].

Thus, while the number of patients will continue to increase, the number of physicians and ICU beds are insufficient to keep up with the demand.

In Japan, life-sustaining support for critically ill patients regardless of medical futility was routinely applied until recently. Over the last decade, a series of controversial incidents related to end-of-life issues triggered debate about end-of-life care in Japan and guidelines for end-of-life care have been developed by the Ministry of Health, Labour and Welfare and several major medical societies [6–9]. Despite these guidelines, some physicians practicing in Japan remain concerned about the adverse legal ramifications believing that guidelines offer no legal protection concerning decisions to withdraw or withhold care. These issues are not well known outside of Japan.

In this review, we discuss the history and issues surrounding end-of-life care in Japan vs. the USA and other Asian countries. The current practice of end-of-life care in the ICU setting will be discussed including efforts by the Japanese Society of Intensive Care Medicine (JSICM) to advance end-of-life care in Japan and future directions in end-of-life care in Japan.

## Review

### History of end-of-life care in the USA

The concept of the living will was introduced in 1969 by Luis Kutner, an Illinois lawyer [10], and it triggered discussions about end-of-life care in the USA (Table 2) [11, 12]. The case of Karen Quinlan in 1976 motivated California to enact the Natural Death Act which was the world's first law allowing withdrawal of life-sustaining support. Following several important US court cases including Barber vs. Superior Court (1983), the case of Claire Conroy (1985), and Cruzan vs. the Missouri Department of Health (1990) established the patients' right to refuse treatment and allow death under certain conditions, if they have the inability to consent.

**Table 2:** **Major historical events in end-of-life care in the USA** [11,12]

Year	Name of case	Issues	Importance
1914	Schloendorff vs. Society of New York Hospitals	Surgical intervention was performed despite withholding of consent by a patient	Competent patients have a right to determine their therapeutic intervention and informed consent is required before the intervention
1969			The concept of living will was introduced by Luis Kutner, an Illinois lawyer
1976	Karen Quinlan	Withdrawal of mechanical ventilation from a patient in a vegetative state	Competent patients have a right to refuse interventions, and this can be applied by surrogates under the principle of substantial judgment if a patient becomes incompetent
1976	California State	-	The Natural Death Act allowed withdrawal of life-sustaining support
1983	Barber vs. Superior Court	Withdrawal of mechanical ventilation from a vegetative patient at the request of the patient's family	Surrogates can refuse interventions on behalf of patients, based on the patient's best interests
1985	Claire Conroy	The right of a patients' guardian to stop artificial nutrition	Treatment that does not benefit or causes harm to the patient should not be continued based on humanitarian reasons in situations where the patient's wishes for end-of-life care are not known
1990	Cruzan vs. the Missouri Department of Health	Withdrawal of care from an incompetent patient who had prior wishes	States can set the level of evidence required to determine the prior wishes of incompetent patients with which surrogate decisions are made.
1990	US government	-	The Patient Self-Determination Act obligated medical insurance organization and medical facilities to update the status of advance directives in patient's medical charts on admission to hospice, hospital or nursing home
2005	Terri Schiavo case	How to define family and how to proceed with end-of-life care decisions if members of the immediate family are not in agreement	The US District Court in Florida denied the emergency request signed by the US President to reinsert the feeding tube

In 1990, the US government enacted the Patient Self-Determination Act [13] and obligated medical insurance organizations and medical facilities to update the status of advance directives in patient's medical charts on admission to hospice, hospital or nursing home. With this act, the concept of advance directives became more widely known in the USA, and by 2007, 41% of the Americans had living wills [14]. However, the living will is not sufficient to completely convey a patient's treatment or care wishes. The variation in values (different races, religions, cultures, ages, etc.) among family or friends, or in the prognosis, further complicates decisions regarding withdrawal of life support or not escalating medical treatment [15–17]. Through the case of Terri Schiavo with no living will in 2005, the nomination of a friend or family member as health care proxy or durable power of attorney is considered a requisite complement to the living will [12]. The advance directive combining living wills and either durable power of attorney or health care proxy was initially introduced in Florida in 1996. It was well received, and later introduced at the national level. This is now known as the ‘Five Wishes Advance Directive’ (Table 3) and has been adopted in the 42 states [18].

**Table 3:** **Five wishes advance directive (adopted by 42 states in the USA)**[18]

	Wish
1	The person I want to make care decisions for me when I can't
2	The kind of medical treatment I want or don't want
3	How comfortable I want to be
4	How I want people to treat me
5	What I want my loved ones to know

### History of end-of-life care in Japan

The first time that the end-of-life care received notable attention in Japan was with the Tokai University Hospital case in 1991 (Table 4) [19, 20]. In this trial, three criteria to justify the withdrawal or withholding of care were clarified, but decision making by a surrogate was not legally accepted. Also, this ruling established euthanasia as illegal in Japan. As a result, the physician was found guilty of murder and sentenced to 2 years in prison with 2 years of probation.

**Table 4:** **Major historical events in end-of-life care in Japan**[19, 20]

Year	Name of hospital	Issues	Importance
1991	Tokai University Hospital	Administration of potassium chloride to ease a patient's respiratory discomfort at the request of the patient's family	Withholding or withdrawal of care is applied when the following condition fits:
			1. A patient is terminally ill and death is inevitable
			2. Living will or advance directive exists at the time of end-of-life decision
			3. Mechanical ventilation, dialysis, medication, blood transfusion, medication, and artificial nutrition are targets of withholding or withdrawal of care
		A living will or an advance directive by the patient did not exist and the surrogate decision in the setting of poor understanding of the patient's condition was not appropriate to determine the patient's end-of-life care	Euthanasia was established to be illegal. A physician was sentenced 2 years in prison
1998	Kawasaki Kyodo Hospital	Withdrawal of endotracheal tube and administration of neuromuscular blockade for respiratory discomfort	Withdrawal of care in this situation was not legally indicated; however, the Supreme Court did not cite a specific norm when withholding or withdrawal of care is applied
		Confirming poor recovery or prognosis 2 weeks after the insult was too early without clinical evidence (e.g., electroencephalogram)	A physician was sentenced 1 1/2 years in prison
		Withdrawal of care requested by the patient's family was performed without providing appropriate information about the patient's prognosis	
2004	Hokkaido Haboro Hospital	Withdrawal of mechanical ventilation without confirmation of brain death or informed consent	The physician was not prosecuted because subsequent investigation concluded that the patient would have died shortly even with ventilator support
2006	Imizu Civil Hospital	Seven deaths after withdrawal of mechanical ventilation was investigated	The physician was not prosecuted because of insufficient evidence to prove the relationship between ventilator withdrawal and the patients' deaths
2006	Wakayama Medical University Hospital	Withdrawal of mechanical ventilation from a brain-dead patient at the request of the patient's family	Physicians and the hospital were exempted from prosecution
2006-2009	-	-	End-of-life care guidelines were developed by a variety of societies
2009	Fukuoka University Hospital	Withdrawal of percutaneous cardiopulmonary support (PCPS) as requested by the patient's family	The treatment team withdrew PCPS according to the 2007 end-of-life guideline and the team was not prosecuted

Another significant case at Kawasaki Kyodo Hospital in 1998 raised several end-of-life care issues previously not discussed in Japan [19, 21]. First, the validity of the diagnosis made by a physician was challenged by a judge that stated it premature to deem the patient at the ‘end-of-life’ after only 2 weeks postcardiac arrest and debated the evidence to support the end-of-life diagnosis. Second, the withdrawal of care requested by the patient's family was questioned because it was performed without confirmation of the patient's wishes or advance directive or obtaining informed consent from the family. The Supreme Court concluded that withdrawal of care without meeting these criteria was legally unacceptable, however, they did not provide criteria in determining whether withholding or withdrawal of care is acceptable. Although the physician denied intent to murder throughout the trial, the appeal was rejected, and the physician was sentenced to a 1 1/2-year prison term and 3 years' probation. This case was sensationalized by the media, and the public misrecognized terminal extubation as an act of murder even though it was described as one of the targets of withdrawal of care in the Tokai University Hospital case and also it had been commonly taking place as a part of end-of-life care in the USA.

Through this case, it became evident that legal provisions and general recognition surrounding end-of-life care in Japan lagged behind other developed countries. Fortunately, no malpractice suits surrounding end-of-life care have occurred since then, but several incidents related to withdrawal of mechanical ventilation have been covered by the media (Table 4).

In 2004, the physician at Hokkaido Prefectural Haboro Hospital withdrew mechanical ventilation from a patient with a diagnosis of brain death who was resuscitated from cardiopulmonary arrest, but did not regain spontaneous breathing [19]. The physician’s behavior was later questioned because the diagnosis of brain death was made by a single physician. Also, withdrawal of mechanical ventilation without providing information to the family or obtaining their consent prior to the act was questioned. However, subsequent investigation concluded that the patient would have died shortly thereafter even with support. As the causal relationship between death and withdrawal of mechanical ventilation was not confirmed, the physician was not prosecuted.

In 2006 the Toyama Prefectural Police investigated a surgical director at Imizu Civil Hospital regarding seven deaths between 2000 and 2005 after withdrawal of mechanical ventilation [19]. There was insufficient evidence to prove the relationship between the director's act and the patients' death, hence he was not prosecuted.

At Wakayama Medical University Hospital in the same year, mechanical ventilation was withdrawn per the family's request from an 88-year-old woman who became brain-dead following an emergency craniotomy for cerebral hemorrhage [19]. The hospital notified the police of her death as unexpected; however, the physicians and the hospital were exempted from prosecution.

Why were the physicians in the first two cases prosecuted, but the physicians in the latter three cases not prosecuted? A possible explanation is the administration of potassium chloride in the first case, and neuromuscular blockade in the second case both of which directly led to death in a short period of time. The cases where physicians were not prosecuted involved only the withdrawal of mechanical ventilation as part of providing comfort care. In addition, the causal correlation between withdrawal of mechanical ventilation and death is indirect and subject to interpretation.

These incidents spurred the creation of end-of-life care guidelines by various Japanese medical societies (Table 5) [6–9].

**Table 5:** **Major end-of-life guidelines in ICU setting in Japan**

Date of issue	Society	Pattern	Contents
August 2006	The Japanese Society of Intensive Care Medicine	Acute: critical care	Nature of terminal care of critically ill patients in intensive care
May 2007	The Japanese Ministry of Health, Labour and Welfare	General	Decision-making process of end-of-life care
November 2007	The Japanese Association for Acute Medicine	Acute: emergency	End-of-life care in the setting of emergency medicine
2010	The Japanese Circulation Society	Chronic: cardiac	Statement for end-stage cardiovascular care

### Guidelines for end-of-life care in Japan

The JSICM published the first guideline addressing end-of-life care in Japan entitled ‘Nature of Terminal Care of Critically Ill Patients in Intensive Care’ in 2006 [6]. The following year in May 2007, the Japanese Ministry of Health, Labour and Welfare issued ‘Guidelines for Decision-Making Process of End-of-Life Care’. Their guidelines addressed the following three points:

1. End-of-life care decisions should be based on informed decision making by the patient or family health care proxy with information provided by the physician(s).
2. Withdrawal of aggressive treatment should be determined by a health care team.
3. The importance of relieving discomfort and pain sufficiently, while also providing comprehensive, medical care including mental and, social support to patient and family by the healthcare team [7].

The Japanese Association for Acute Medicine (JAAM) issued the ‘Recommendations for End-of-Life Care in Emergency Medicine’ in November 2007 [8]. They defined end-of-life as existing when any of the following situations are present: (1) irreversible brain dysfunction; (2) an artificial device is necessary to support life with no alternative means, such as transplant, to reverse organ dysfunction; (3) death anticipated within a few days even with current treatment continued; and (4) end-stage disease (e.g., cancer) when the diagnosis is made after the initiation of treatment. JAAM also recommended that more than one physician should participate in the determination of end-of-life.

The Japanese Circulation Society issued ‘Statement for end-stage cardiovascular care’ in 2010 [9] and addressed the importance of comprehensive supportive care for the patients and their family in the setting of end-stage cardiovascular diseases including chronic heart failure, arrhythmia, chronic kidney disease, and stroke.

### The current status of end-of-life care in Japan

In 2009, a 68-year-old man was brought into Fukuoka University Hospital for severe respiratory failure requiring mechanical ventilation and percutaneous cardiopulmonary support (PCPS) [22]. Three weeks after admission, the treatment team withdrew PCPS based on the patient's prior wishes and the JAAM end-of-life guidelines [8]. This case drew intense media attention; however, the treatment team was not subsequently prosecuted, giving reassurance to Japanese physicians that they could safely practice end-of-life care.

Subsequent to these incidents, a 2009 survey reported that 90% of medical professionals and 80% of the public were interested in end-of-life care issues [23]. More than 90% of the public respondents wished to receive a direct notification from a physician if the time of death was approaching. The proportion of people who did not want life-sustaining treatment or cardiopulmonary resuscitation (CPR) was 73%, which increased compared to a previous survey [24]. Patients are beginning to assume a greater role in determining end-of-life care decisions. However, some issues still remain to be addressed.

First, physicians in Japan are still reluctant to withhold or withdraw life-sustaining support although end-of-life guidelines support it. According to a recent survey by JSICM [25], withholding and withdrawal of mechanical ventilation from terminally ill patients were practiced in approximately 45% and 10%, respectively.

Second, the concept of end-of-life care has not been widely prevalent even after the publication of guidelines. According to a brief survey by the Japanese Society of Physicians and Trainees in Intensive Care (JSEPTIC), the percentage of younger physicians (within 10 years after graduation) who were familiar with the end-of-life care guidelines was only 58% for the JAAM guideline, followed by 40% or less for other guidelines [26]. Surprisingly, up to 27% of these physicians did not know any of the guidelines. One can assume that despite the recent media attention, the general public would be even less cognizant about end-of-life policies.

Third, documentation and legislation of a living will and advance directives have not been widely accepted among the public yet.

Akabayashi et al. in 2003 did a survey investigating awareness about end-of-life issues among the public [27]. Of 425 layperson citizens living in Tokyo, 80.5% of the respondents desired disclosure of prognosis and treatment if they were in an end-of-life situation. Despite this, only 48.5% of the respondents requested that the advance directive be documented in writing, and the remaining 42.4% of respondents preferred to convey their treatment policy to their doctor or family verbally, without documentation. Such a preference has been common in East Asian countries for many years [28, 29]. According to the latest questionnaire by the Japanese Ministry of Health, Labour and Welfare, even though 70% of the general public and 74% of physicians supported a living will, only 22% of the general public and 16% of physicians agreed with the legislation of a living will [30].

### End-of-life care in Japan in comparison with other countries

Yaguchi et al. in 2005 conducted a survey of 1,961 intensivists in 21 different countries, including Japan, about end-of-life care decision making [31]. In this study, the proportion of physicians who determined the treatment plan in discussion with nurses was lower in Japan (39%) compared to Northern and Central European countries (62%). In addition, only one third of the Japanese physicians obtained a do not attempt resuscitation (DNAR) order in an end-of-life situation. This proportion was much lower than in Northern and Central Europe, North America, and Australia where more than 80% of physicians applied written DNAR orders. Almost half of Japanese physicians continued full support in vegetative patients that developed septic shock. This proportion was significantly higher than in Europe and Australia where less than 10% of physicians agreed with full support for such situation. A similar result was reported by Asai et al. in 1999 where attitudes of Japanese physicians who were involved in managing persistent vegetative patients were investigated [32]. Of 190 respondents, only 30% agreed in withholding of antibiotics when the patient developed pneumonia. Why do Japanese physicians continue treatment for vegetative patients? First, life and this world have been highly valued and death has been typically denied and abhorred among Japanese people [32]. Second, providing the best possible care at any stage of a disease has been considered to be in the best interest of the patient among Japanese physicians [33]. Third, physicians fear lawsuits when they withhold or withdraw life-sustaining equipment or devices from such patients [34]. Fourth, making treatment plans by discussion between physicians and the patient's family is a traditional and common style seen in East Asian countries such as China, South Korea, and Japan [27–29]. In addition, the patient's family generally requests continuing of the current treatment, and it is difficult for physicians to decline the offer. One study showed that Japanese Americans tend to make treatment decisions on their own, as compared to Japanese respondents [16]. However, both groups still sought decision-making by a group involving the healthcare proxy. This may be influenced by Confucianism that has been present in these countries for centuries. However, the degree of influence of Confucianism appears to be different among different countries.

In China where Confucianism still has a major impact, discussions related to death and end-of-life care are avoided, and the acceptance of end-of-life care among the public is quite low [35]. Confucianism gives filial piety to parents and these values may lead to aggressive treatment even if the parent is in the terminal stage of disease. In a study in China, 72% of Chinese physicians confessed difficulty suggesting to a patient's family about limiting life-sustaining therapy [28]. Furthermore, up to 53% of Chinese physicians opposed obtaining DNAR from end-of-life patients while only 5% of European physicians were opposed.

Confucianism also has had a big impact on South Korea, influencing treatment decisions made by families even if the patient has decision-making capacity [29, 36]. Two major incidents in Korea over end-of-life care resulted in the Korean Association of Medical Societies (KAMS) to issue guidelines for end-of-life care in 2002 [37] and in 2009 [38] in response to these incidents, and Korean people have become more accepting of their involvement in their own end-of-life care. In a survey of 382 civilians in 2011, 92% of respondents agreed with the creation of an advance directive. They supported advance directives because they were in favor of dignified death without life-sustaining support at end-of-life and also knew that families do not function well in the end-of-life period [29]. There is no legislation to date in China or South Korea granting legal approval to withhold life-sustaining support. This is in contrast to Thailand where the right to make decisions about end-of-life care was enacted as the National Health Act of 2007, and the legislation to comply with the law was enacted in 2012 [39].

In Japan, family-centered decision making at the end-of-life has been preferred as in China and South Korea [16, 27, 40]. This is likely because interdependence and harmony that are addressed in Confucianism have great significance as social values for Japanese people [41, 42]. This might make it more difficult for Japanese people to accept the concept of a living will and advance directives which are generally made by the patient.

### Efforts by the Japanese society of intensive care medicine

JSICM issued new goals in 2009 based on several current end-of-life guidelines [43]. These include the following: (1) establishing and expanding a support system for physicians involved with end-of-life care, (2) collecting end-of-life cases and sharing the information in order to standardize medical judgment, (3) establishment of spiritual care for patients and families, (4) proposal and dissemination of information about end-of-life care to the public. In addition, the JSICM invited expert clinical ethicists to establish a system for end-of-life care [44]. Since these goals were issued, a total of four 24-h educational courses on clinical ethical issues in end-of-life care were held in 2011 [44]. Also, a total of three 3-day courses of ‘spiritual care for family of end-of-life patients in ICU’ were held including core didactics and the use of role play [44]. Currently, the JSICM is working with JAAM to create a single guideline for end-of-life care in the setting of ICU [45] and is also working to educate the public about end-of-life concepts.

## Conclusion

The acceptance of palliative care at the end-of-life has advanced greatly in Japan during the last 8 years moving from an uncertain and threatening environment where withholding or withdrawal of care might lead to criminal prosecution to recognition of the importance of the decision-making process, guideline-based management, and education of both physicians and the public in end-of-life care.
